# Secretory Patterns in Colleters of Apocynaceae

**DOI:** 10.3390/plants10122770

**Published:** 2021-12-15

**Authors:** Josiana Cristina Ribeiro, Elisabeth Dantas Tölke, Diego Demarco

**Affiliations:** Departamento de Botânica, Instituto de Biociências, Universidade de São Paulo, Sao Paulo 05508-090, Brazil; josicrisrib@gmail.com (J.C.R.); elisabeth.tolke@gmail.com (E.D.T.)

**Keywords:** foliar colleters, cytochemistry, secretory process, release mode, Apocynaceae

## Abstract

Colleters of Apocynaceae are glands related to different types of protection of vegetative and floral meristems through the production of mucilage or a mixture of many different compounds. Although several anatomical papers have shown histological and histochemical aspects of colleters of the family, almost nothing is known about their secretory process. In this study, we analyzed two types of colleters in Apocynaceae: one produces mucilage and lipophilic compounds, while the other produces an exclusively mucilaginous secretion. The secretory epidermis of the colleters of *Allamanda schottii* and *Blepharodon bicuspidatum* has a dense cytoplasm with organelles responsible for the production of mucilage and lipids. This heterogeneous secretion is released through granulocrine and eccrine mechanisms and is temporarily stored in a subcuticular space before crossing the cuticle. Conversely, colleters of *Mandevilla splendens* and *Peplonia axillaris* produce only mucilage and have a very different secretory apparatus. The mechanism of secretion is granulocrine, and the exudate is firstly accumulated in a large periplasmic space and later in an intramural space before crossing the cuticle. Notably, the structure of the cuticle varies according to the secretion composition. Although the colleters of the family are histologically similar, this study demonstrates a metabolic and subcellular variability previously unknown for Apocynaceae.

## 1. Introduction

Colleters are glands related to protection of meristems [1], which are widespread in Apocynaceae [2], occurring on the lamina and petiole of leaves, cotyledons, bracts, bracteoles, calyces, and corollas [1,3,4,5,6]. These glands have taxonomic importance for the family [6], and some of those found laterally to the base of leaves and in interpetiolar position have stipular origin, related to the evolution of stipules in Gentianales [7].

Morphologically, colleters of Apocynaceae have usually been classified as the standard type, being cylindrical or dorso-ventrally flattened, formed by a secretory head and a stalk or by just the secretory portion [1,6]. The secretory tissue of colleters is exclusively epidermal, which produces a viscous secretion that may be composed of mucilage, resin [1], or a mixture of mucilage and lipophilic substances [1,6,8,9,10]. Histochemical studies have demonstrated that the colleters of Apocynaceae secrete mainly mucilage and lipids, which involve the meristems and developing organs, protecting them against desiccation, fungi, and/or small phytophagous insects [10].

The colleter secretion is accumulated in a periplasmic space before being released to the outside through the wall and the cuticle without breaking it [5,6,11]. However, the release of secretion through rupture of the cuticle has already been reported for the family [12,13,14,15], and there have been reports of separation of cells due to the dissolution of the middle lamella related to secretion mechanism [16,17,18]. In *Tabernaemontana catharinensis*, the only species of Apocynaceae ultrastructurally analyzed to date, evidence of eccrine and granulocrine secretion types was observed [5], reinforcing the need for further studies in the family to verify the various aspects of the secretory process in other species.

Considering that some species have colleters whose secretion is heterogeneous, composed of mucilage and lipophilic substances (e.g., *Allamanda* and *Blepharodon*), and others have exclusively mucilaginous secretion (e.g., *Mandevilla* and *Peplonia*) [10], an analysis of the secretory mechanism of the different colleters is relevant to understand the evolution of the gland in this family since different types of compounds may be produced in distinct organelles and released by varied pathways [8]. Due to the lack of ultrastructural studies in the family demonstrating the production mode of the secretion and its release to the outside, our goal was to describe the secretory cells of colleters in four species of Apocynaceae with different secretion composition. We also performed the first comparative analysis of the subcellular secretory mechanisms in relation to the secretory metabolites in colleters.

## 2. Results

**Figure 1:**
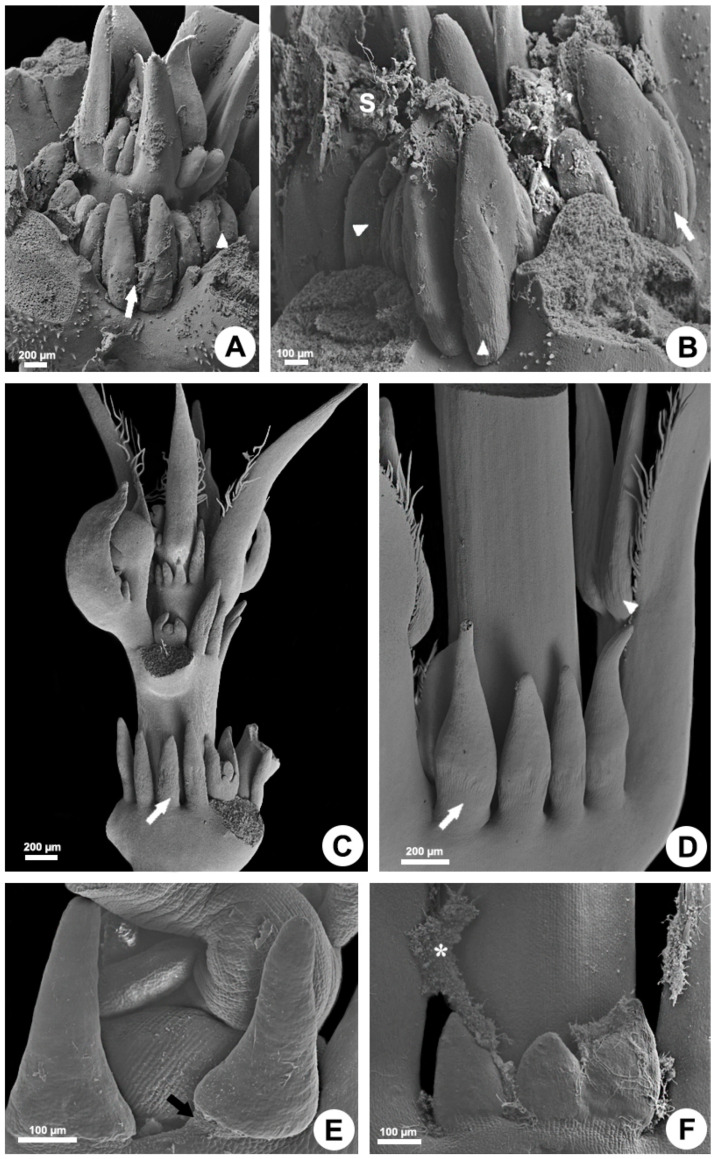
Morphology of the colleters of *Allamanda schottii* (**A**,**B**), *Blepharodon bicuspidatum* (**C**,**D**), *Mandevilla splendens* (**E**), and *Peplonia axillaris* (**F**). Scanning electron microscopy. White arrows = interpetiolar colleters; Black arrow = colleter stalk; Triangle = petiolar colleter; Asterisk = fungi; S = secretion.

**Figure 2:**
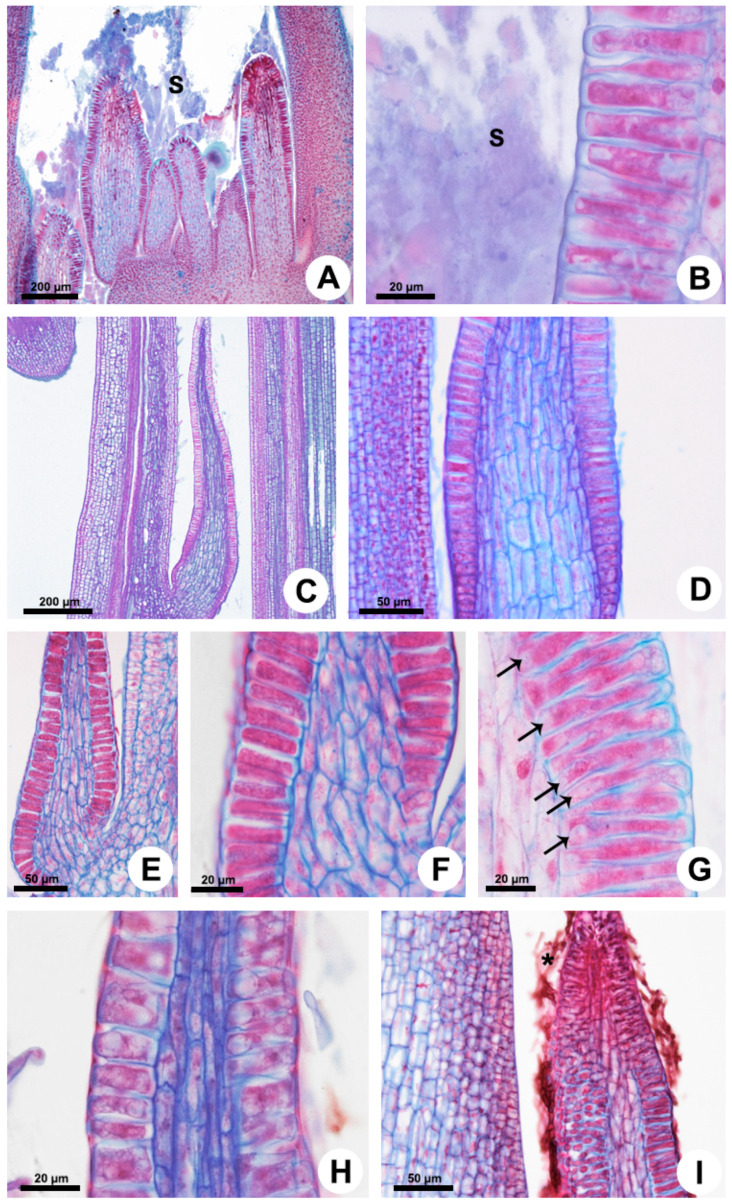
Anatomy of the colleters of *Allamanda shottii* (**A**,**B**), *Blepharodon bicuspidatum* (**C**,**D**,**G**,**H**), and *Peplonia axillaris* (**E**,**F**,**I**). Light microscopy. Longitudinal sections. (**A**,**C**,**E**,**H**) General view. (**B**,**D**,**F**,**G**,**I**) Detail of the secretory epidermis covering a parenchyma core. Note the presence of small vacuoles in the secretory cells (**G**) and the absence of vasculature. (**I**) Fungal hyphae (asterisk) on the mucilage colleter. Arrow = vacuole; S = secretion.

In all the analyzed species, the foliar colleters have similar morphology and anatomy, with a notable absence of vasculature, and vary only in relation to the presence of a stalk (Figure 1 and Figure 2). They are located in the shoot apices and are easily observable to the naked eye in the nodal regions, occupying petiolar and interpetiolar positions. The interpetiolar ones occur on the sides of the petioles in *Allamanda* (Figure 1A,B) and are distributed continuously between the petioles in *Blepharodon*, *Mandevilla*, and *Peplonia* (Figure 1C–F). The petiolar colleters are located at the base of the petioles, close to leaf axile in *Allamanda* (Figure 1A,B) and *Mandevilla*, or in the distal portion of the petiole close to the leaf blade in *Blepharodon* (Figure 1D) and *Peplonia*.

### 2.1. Morphology and Anatomy

Foliar colleters are conical, rarely bifid and deciduous (Figure 1D). They have a broad base in *Allamanda* (Figure 1A,B) and in *Peplonia* (Figure 1F) and are rectilinear in *Blepharodon* and *Mandevilla* (Figure 1C–E). Morphologically, the colleters can be of two types, stalked or sessile. *Allamanda* (Figure 2A), *Blepharodon* (Figure 2C), and *Mandevilla* (Figure 1E) have stalked colleters, and *Peplonia* has sessile ones (Figure 2E).

Anatomically, the stalked colleters present a non-secretory basal portion (Figure 2A), while the sessile ones are completely coated by a secretory epidermis (Figure 2E). In all species, the secretory portion of the colleters present a non-secretory parenchyma axis devoid of vascularization covered by a uniseriate secretory epidermis in palisade (Figure 2D–G). Epidermal cells have thin walls and cuticle, dense cytoplasm (Figure 2B,F,G), and vacuoles of various sizes (Figure 2G,H). Cells in the early stage of the secretory process have vacuoles occupying the basal portion of the cells (Figure 2G). Those in the final phase of the secretion present one large central vacuole occupying most of the cell lumen, as well as a nucleus with a conspicuous nucleolus (Figure 2H). The colleters secrete a viscous white exudate, which permeates the entire shoot apex (Figure 1B and Figure 2A,B). No fungal proliferation was observed in *Allamanda* and *Blepharodon* (Figure 1A–D and Figure 2A–D), but a large amount of hyphae was recorded in *Peplonia* (Figure 1F and Figure 2I). All colleters of each species produce the same type of secretion during the entire secretory activity.

### 2.2. Ultrastructure

#### 2.2.1. Colleters with Mucilaginous Secretion (*Mandevilla splendens* and *Peplonia axillaris*)

- Cell wall and cuticle

**Figure 3:**
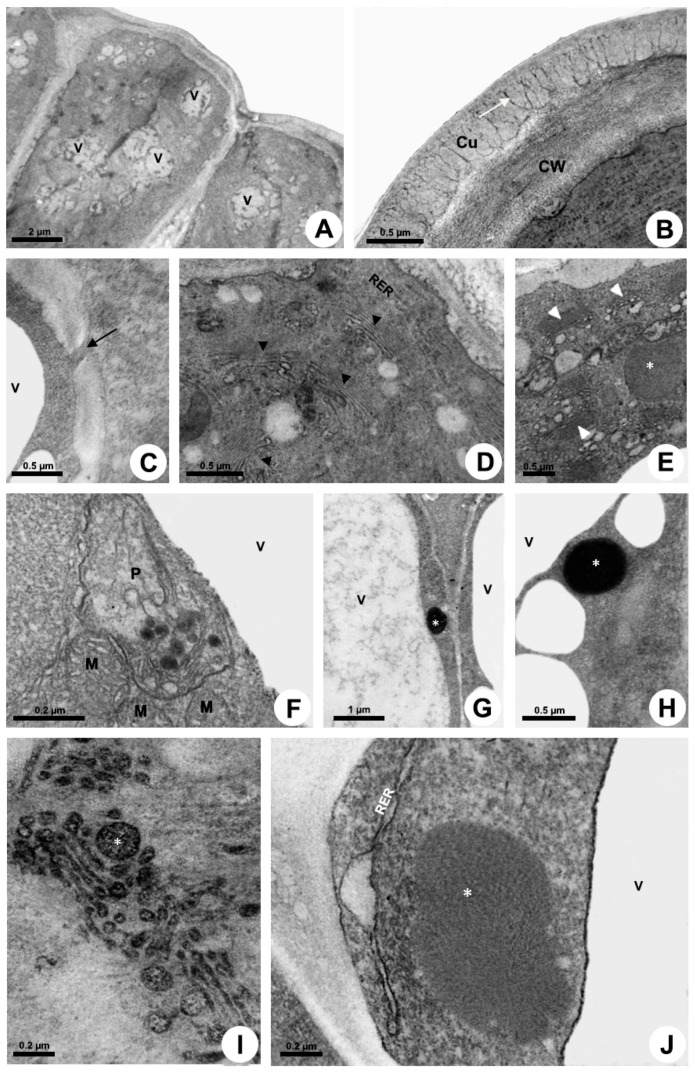
Ultrastructure of the colleter secretory cells of *Mandevilla splendens* (**A**–**H**,**J**) and *Peplonia axillaris* (**I**). Transmission electron microscopy. (**A**) General view. (**B**) Cuticle (Cu) with pectin projections (arrow) from the cell wall (CW). (**C**) Plasmodesma (arrow). (**D**,**E**,**G**–**J**) Abundant dictyosomes (arrowhead) and RER in parietal position. Note vesicles filled with mucilage (asterisk). (**F**) Plastid (P) with plastoglobules surrounded by many mitochondria (M). (**G**,**H**) Detection of polysaccharides within vesicles with PATAg test (asterisk). (**I**) Detection of mucilage in the dictyosome vesicles using ruthenium red test (asterisk). (**J**) Beginning of a vesicle formation from RER close to plasma membrane.

The secretory cells have thin walls and cuticle (Figure 3A), with a great amount of pectin projections observed from the outer periclinal cell wall inwards, almost the entire cuticle (Figure 3B). The anticlinal walls present a large amount of plasmodesmata, facilitating the exchange of material between the secretory cells (Figure 3C).

- Cytoplasm

Epidermal cells have a dense cytoplasm rich in ribosomes with an extensive network of rough endoplasmic reticulum (RER) (Figure 3D), a profusion of hyperactive dictyosomes (Figure 3D,E), large mitochondria, vacuome, and many scattered vesicles (Figure 3A,D–J). Plastids were rarely found (Figure 3F). A large amount of mucilage within the vacuole and vesicles was recorded (Figure 3E,G–J). The abundance of vesicles filled with secretion on the trans face of the dictyosomes indicates a large production of mucilage (Figure 3I) which is transferred to the RER, which is mainly associated with the dictyosomes in a peripheral position, close to the plasma membrane in the distal portion of the cell (Figure 3D,J). The secretion produced in the proximal (basal) portion of the cell is temporarily stored in the vacuole (Figure 3G) and released later in the distal portion through emission of vesicles (Figure 3A).

- Secretion release

**Figure 4:**
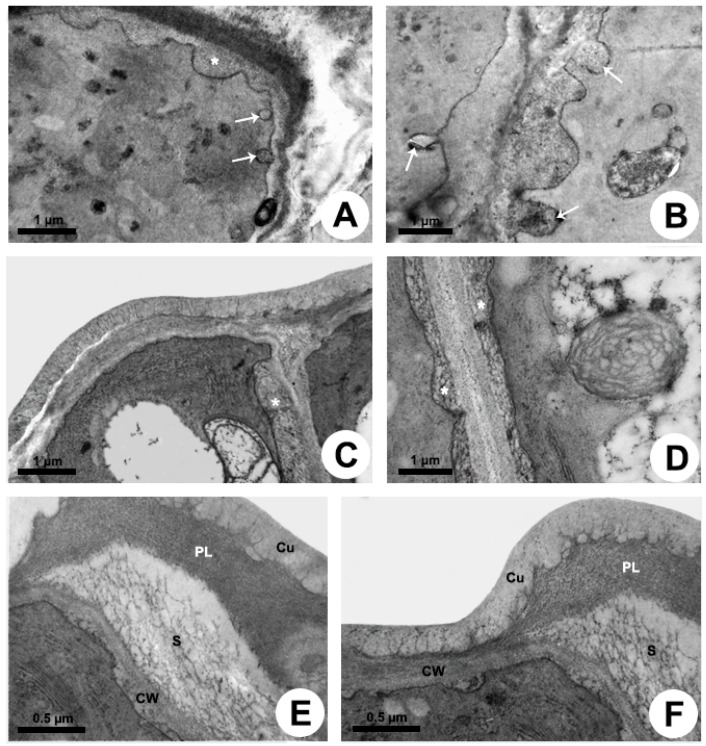
Secretion release in colleters of *Peplonia axillaris* (**A**,**B**) and *Mandevilla splendens* (**C**–**F**). Transmission electron microscopy. (**A**–**D**) Fusion of vesicles with plasma membrane and formation of a large periplasmic space. (**E**,**F**) Intramural space filled with secretion. Arrow = merging vesicle with plasma membrane; Asterisk = periplasmic space with secretion; Cu = cuticle; CW = cell wall; PL = pectin-rich layer; S = secretion.

The release mechanism is granulocrine. Fusion of vesicles and small vacuoles with the plasma membrane is observed in the apical region of the cell, releasing the secretion outside the protoplast close to the outer periclinal cell wall and to the distal portion of the anticlinal wall (Figure 4A,B). After the fusion of vesicles with the plasma membrane, the secretion is transferred to a large periplasmic space before crossing the cell wall and cuticle (Figure 4A) or before moving to the adjacent cell (Figure 4B–D). During the passage of the secretion through the outer periclinal cell wall, a cavity is formed within the wall, here named “intramural space”, placed beneath the pectin layer in the outermost portion of the wall (Figure 4E,F). Thus, there are two sites of temporary extraprotoplastic storage of the secretion before its release to the surface of the gland: one periplasmic and the other intramural which is located between the cellulosic portion of the wall and the pectin layer. After completely crossing the wall, the secretion passes through the cuticle without breaking it. Initially, the mucilage is conducted by long, branched projections of pectin within the cuticular layer, which almost reach the surface, and then through a very thin cuticle proper (Figure 3B and Figure 4E,F), being released to outside.

#### 2.2.2. Colleters with Heterogeneous Secretion (*Allamanda schottii* and *Blepharodon bicuspidatum*)

- Cell wall and cuticle

**Figure 5:**
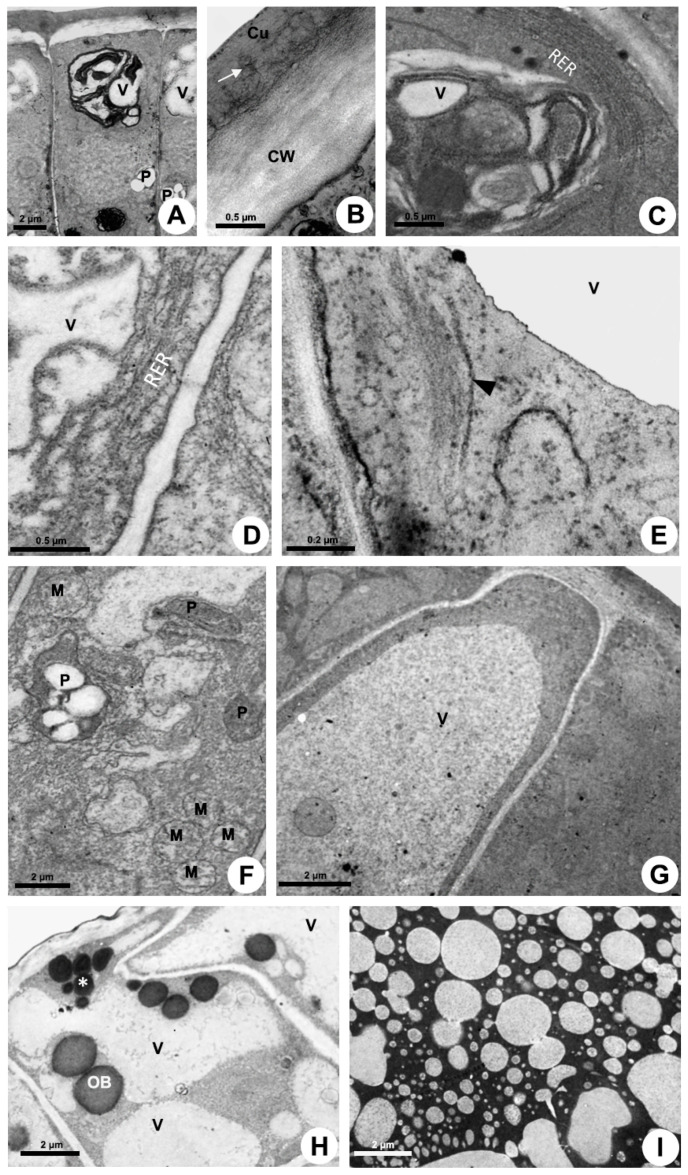
Ultrastructure of the colleter secretory cells of *Blepharodon bicuspidatum* (**A**,**B**,**D**–**F**,**H**) and *Allamanda shottii* (**C**,**G**,**I**). Transmission electron microscopy. (**A**,**C**) Vacuoles with heterogenous content. (**B**) Pectin projections from the cell wall (CW) into the cuticle (Cu). (**D**) RER in parietal position. (**E**) Dictyosome vesicles being directed to the plasma membrane. (**F**) Plastids with starch grains and mitochondria. (**G**) Central vacuole filled with secretion. (**H**) Vesicles with polysaccharides detected using PATAg test (asterisk) and oil bodies (OB). (**I**) Detection of lipids with imidazole-osmium tetroxide test in the secretion on the colleter. Arrow = pectin projections within cuticle; Arrowhead = dictyosome; M = mitochondrion; P = plastid; RER = rough endoplasmic reticulum; V = vacuole.

The walls of the secretory cells are usually thin and covered by a thin cuticle (Figure 5A), which has several projections of pectin extending inwards its basal half (Figure 5B). Plasmodesmata were not observed between the epidermal cells.

- Cytoplasm

**Figure 6:**
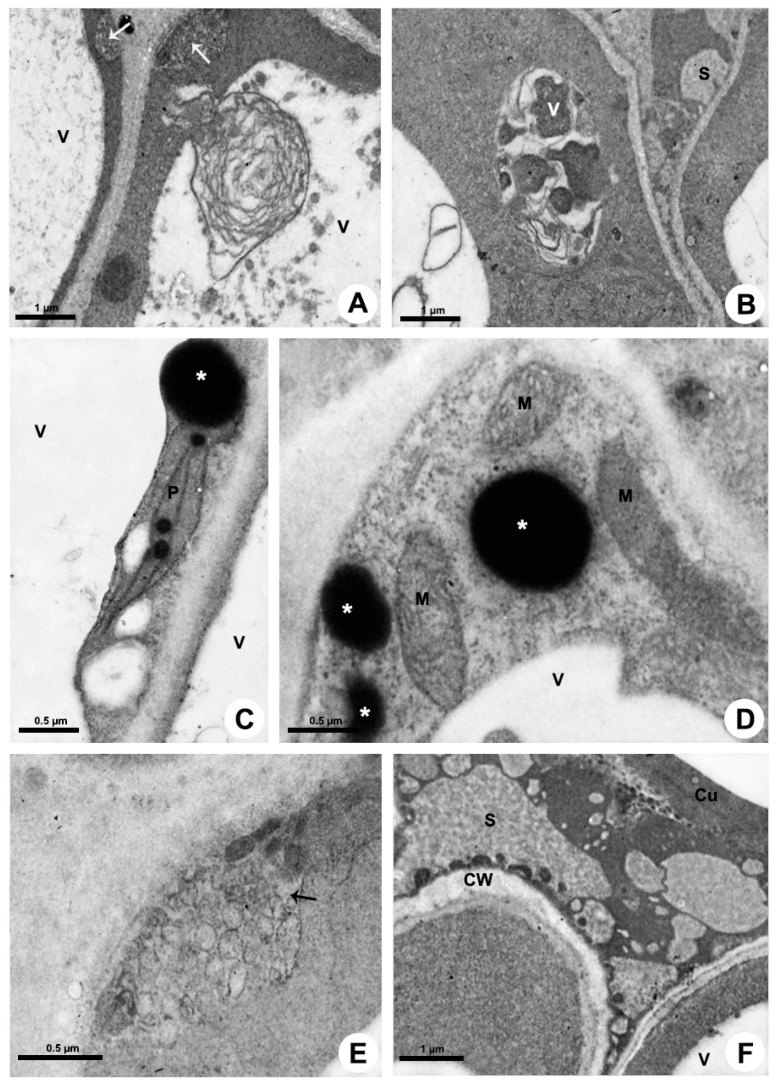
Secretion release in colleters of *Allamanda shottii* (**A**,**B**,**E**,**F**) and *Blepharodon bicuspidatum* (**C**,**D**). Transmission electron microscopy. (**A**,**E**) Vesicles newly fused with plasma membrane transferring the secretion to the periplasmic space (arrow). (**B**) Small vacuole with heterogenous secretion. (**C**,**D**) PATAg test evidencing polysaccharides in vesicles (asterisk). Note plastid (P) with starch grains and plastoglobules, and mitochondria (M). (**F**) Heterogenous secretion in the subcuticular space. Cu = cuticle; CW = cell wall; S = secretion; V= vacuole.

The colleters have a dense cytoplasm rich in ribosomes (Figure 5 and Figure 6). The RER is prominent and is mostly located in parietal position (Figure 5C,D); dictyosomes were also seen close to the membrane (Figure 5E). There are many plastids with both plastoglobules and starch grains (Figure 5A,F and Figure 6C) and mitochondria (Figure 5A,F and Figure 6D). Vacuoles and vesicles of various sizes with fibrillar osmiophilic material and granular material occupy most of the protoplast (Figure 5A,C,G,H and Figure 6A,B). A large number of vesicles containing mucilage and oil bodies was observed in *Blepharodon* (Figure 5H). After production of mucilage from dictyosomes, the vesicles are directly transported to the distal portion of the cell to be released (Figure 5H) or may be temporarily stored inside the vacuole (Figure 5G) when produced in the proximal portion of the cell. Similarly, oil bodies produced in the plastids and found free in the cytosol (Figure 5H) may be directly released or temporarily stored in the vacuole (Figure 5G). *Allamanda* secretes a much larger amount of lipids (Figure 5I) than *Blepharodon*.

- Secretion release

The release mechanism is mainly granulocrine. Several vesicles and small vacuoles containing secretion derive from dictyosomes, RER, and also from the central vacuole and move toward the plasma membrane in the distal portion of the cell (Figure 5H and Figure 6A–D). Vesicles originated from dictyosomes are directed to the plasma membrane or to the RER, where proteins are added, and then vesicles formed by the RER are released and fuse with the plasma membrane. A large amount of heterogeneous secretion, containing mucilage and lipids, is transferred to a reduced, restricted periplasmic space close to the outer periclinal wall (Figure 6E). Then, the secretion rapidly crosses the cell wall and is temporarily accumulated in a large subcuticular space (Figure 6F). This space is formed by the detachment of the pectin layer from the cuticle at the outermost portion of the periclinal cell wall. In addition to the granulocrine mechanism, oil bodies freely cross the plasma membrane in an eccrine release mode, being posteriorly accumulated in the subcuticular space (Figure 6F). Despite the large amount of secretion underneath the cuticle applying pressure, no rupture was observed during the secretion pathway to the outside.

## 3. Discussion

Foliar colleters were found in petiolar and interpetiolar regions of the shoot apex, producing the same type of secretion during the entire period of the secretory activity, and two patterns of secretory process and mode of release were described, according to the secretion composition.

The colleters studied here can be described as the standard type, present in most Apocynaceae [1,5,6]. Only one layer of secretory epidermal cells is observed in these colleters [1,5,6,11,18,19,20], which characteristically have thin walls and large vacuoles. However, *Tabernaemontana* has cells with poorly developed vacuoles, and the periclinal cell walls are thicker than the anticlinal ones [5]. This differential thickness may be related to the mode of secretion release [21,22], as discussed below.

### 3.1. Secretory Machinery

#### 3.1.1. Mucilaginous Secretion–*Mandevilla* and *Peplonia*

Colleters of *Mandevilla* and *Peplonia* secrete mucilage and proteins [10]. The predominant organelles observed in the secretory cells are dictyosomes, which produce the mucilage that is transferred to the rough endoplasmic reticulum through vesicles. In the RER, proteins are added to the secretion, and vesicles derived from the RER are transported to the plasma membrane, where the secretion is released to the periplasmic space. Mucilage is the main component of the colleter secretion in Apocynaceae [6] and was detected in foliar colleters of several genera of the family, such as *Allamanda*, *Asclepias*, *Blepharodon*, *Fischeria*, *Forsteronia*, *Matelea*, *Oxypetalum*, *Peplonia*, *Plumeria*, *Prestonia*, *Rauvolfia*, *Roupelia*, and *Tabernaemontana* [5,10,11,16,17,18,23,24,25]. Proteins have also been recorded in the secretion of colleters in *Plumeria* [16], *Allamanda*, *Alstonia* [17,26], *Roupelia* [23], and genera of the subfamily Asclepiadoideae [10].

The close association between rough endoplasmic reticulum and dictyosomes producing vesicles close to the plasma membrane has been noticed in colleters of other genera, such as *Tabernaemontana* in Apocynaceae [5], *Caryocar* in Caryocaraceae [27], *Bathysa, Psychotria, Simira*, and *Warzewiczia* in Rubiaceae [28,29], and *Clusia* in Clusiaceae [30]. This subcellular organization was observed in colleters producing hydrophilic secretions but not in colleters producing lipophilic or mixed secretions in Rubiaceae [29], being characteristic of several mucilage glands and consistent with the synthesis and release of protein/carbohydrate-based mucilage [8]. The abundant presence of vesicles in the epidermis indicates a constant production of exudate since they were also recorded for colleters of all families [5,27,28,29,30,31].

Organelles related to the synthesis of lipophilic compounds were rare or not observed in *Mandevilla* and *Peplonia*. Few plastids occur in the secretory cells, and osmiophilic compounds were restricted to the plastoglobules. Smooth endoplasmic reticulum (SER) was not observed, and the imidazole-osmium tetroxide test did not detect the presence of lipids in the secretion. However, SER occurs in colleters of *Bathysa* [32], *Coccocypselum* and *Tocoyena* [29] in Rubiaceae, which secrete a heterogeneous exudate.

#### 3.1.2. Heterogeneous Secretion–*Allamanda* and *Blepharodon*

Colleters of *Allamanda* and *Blepharodon* produce mucilage, proteins, lipids, and phenolic compounds [9,10]. The synthesis of mucilage and proteins occurs in a similar way to that of *Mandevilla* and *Peplonia*. However, those colleters are distinct from the mucilaginous ones in relation to the vacuome being composed of one large vacuole or many vacuoles of various sizes containing a heterogeneous secretion, in addition to abundant plastids containing starch and plastoglobules. Since SER was not observed in both genera, plastids are likely responsible for the production of lipids in the colleters. Plastids containing starch were also detected inside the secretory cells in *Plumeria* [16], *Allamanda* [17], and *Mandevilla* [18] and in parenchyma cells of *Alstonia* [26] and *Tabernaemontana* [5]. The starch within the plastids serves to produce a nutritional and energy reserve for the production of secretion by epidermal cells.

The transfer of secretion to the vacuole, as an intermediate step of the secretory process, is unusual. Although this step is not obligatory, since part of the secretory vesicles fuses directly with the plasma membrane releasing their contents to the periplasmic space, the vacuole seems to participate as an active organelle in the secretion production of these colleters along the entire secretory phase. Notably, this fusion of secretory vesicles to the vacuole has also been observed in colleters of *Psychotria*, *Simira*, *Warzewiczia* (Rubiaceae), *Copaifera* (Fabaceae), *Tontelea* (Celastraceae), and *Cariniana* (Lecythidaceae) [29,31,33,34]. Thus, the vacuole of these colleters does not seem to simply play a role in packaging and transporting the secretion to out of protoplast but also modifying it, influencing the type of compound released on the colleter surface.

### 3.2. Secretion Release

**Figure 7:**
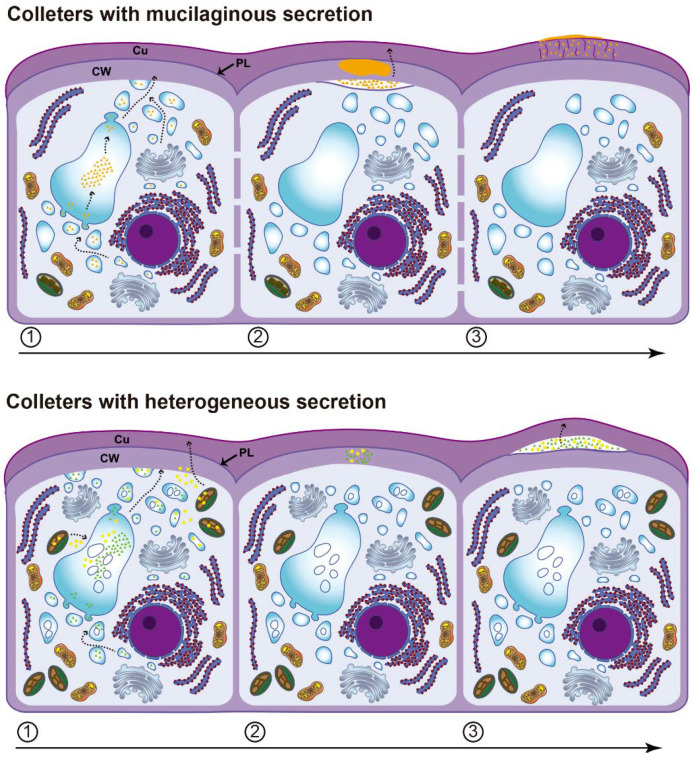
Schematic sequence of three moments of the secretion release in colleters with mucilaginous exudate and those with heterogeneous exudate. Cu = cuticle; CW = cell wall; PL = pectin layer.

The mode of secretion release from the protoplast is mainly granulocrine for all species, but the way it crosses the cell wall and cuticle differs between colleters with mucilaginous secretion and those with heterogenous secretion. Vesicles and small vacuoles fuse to the plasma membrane in the distal portion of the cell, transferring their contents to the periplasmic space. In the mucilaginous colleters, this space is large, and the cell wall represents the first barrier to the release of the secretion to outside (Figure 7).

According to Paiva [35], viscous secretions, such as mucilage, cannot passively cross the cell wall and need to be actively pushed by the protoplast. Although the periplasmic space is not as wide as predicted by the cell cycle proposed by Paiva [35], the need of protoplast to apply pressure to mucilage crosses the wall is evident. During the passage of the secretion within the wall, a second space is formed in the colleters of *Mandevilla* and *Peplonia*, where the mucilage is temporarily accumulated. This intramural space is formed due to the detachment of the pectin layer from the cellulosic portion of the outer periclinal cell wall. Afterward, the mucilage crosses the cuticle through cell wall pectin projections, reaching the colleter surface (Figure 7). The presence of these pectin projections characterizes this portion of the cuticle as the cuticular layer [36], a hydrophilic passage to the mucilaginous secretion of *Mandevilla* and *Peplonia* across the cuticle. The lack of ultrastructural studies likely led most authors to misinterpret these hydrophilic pathways as cuticular “microchannels” [37]. Miguel et al. [21] and Gonçalves et al. [22] observed that the structural organization of the outer periclinal wall of colleters in *Bathysa* and *Prepusa* changes during passage of the secretion, forming a space where the secretion is accumulated. However, this space initially formed in the pectin layer of cell wall, reaches the cuticular layer at maturity [21] and corresponds to the subcuticular space, as observed in *Allamanda* and *Blepharodon*.

Despite the presence of lipids and the higher viscosity of the secretion in colleters of *Allamanda* and *Blepharodon*, the granulocrine process produces small periplasmic spaces, relative to the area of vesicle fusion, which rapidly disappear with the passage of the secretion through the wall (Figure 7). This difference in relation to the mucilage release in colleters of *Mandevilla* and *Peplonia* may indicate a looser arrangement of the pectin in the cell wall, since pectins are the main wall component responsible for its porosity [38]. Although this heterogenous secretion seems to pass freely through the wall, it causes the detachment of the cuticle and is accumulated in a large subcutilar space (Figure 7). The increasing pressure in this space due to the constant addition of more secretion is likely responsible for pressing the exudate through the cuticle, reaching the colleter surface. Remarkably, this action does not rupture the cuticle, which appears to be more permeable to this partially lipophilic secretion. This hypothesis is reinforced by the distinct structure of the cuticle in both types of colleters. In the mucilage colleters of *Mandevilla* and *Peplonia*, the pectin projections of the cell wall extend along almost the entire cuticle. On the other hand, these projections terminate in the middle of cuticle in colleters with heterogeneous secretion in *Allamanda* and *Blepharodon*, i.e., the cuticle proper, which is formed only by lipophilic components [36], corresponding to half of the cuticle thickness.

The granulocrine secretion is the main mechanism of exudation observed in colleters. Secretory vesicles and vacuoles which fuse with plasma membrane releasing the secretion into the periplasmic space occur in all colleters described to date, as observed in Apocynaceae, Bromeliaceae, Caryocaraceae, Celastraceae, Clusiaceae, Euphorbiaceae, Fabaceae, Lecythidaceae, and Rubiaceae [5,27,29,30,31,33,34,39,40,41]. Additionally, some lipids may also be directly released through the plasma membrane without being packed in vesicles (eccrine mechanism) in *Allamanda* and *Blepharodon*. Similarly, eccrine secretion has been observed in colleters of *Tabernaemontana*, *Croton*, and some Rubiaceae [5,29,40].

After leaving the protoplast, the secretion must cross the cell wall and cuticle to reach the colleter surface. The release of secretion to the outside may occur due to the gradient concentration or by an active process [35,42]. Colleters of Apocynaceae exude mucilage or a mixture of mucilage and lipophilic compounds, most of the time without rupturing the cuticle [6]. Therefore, the passage of hydrophilic compounds through a hydrophobic substance (cutin) without breaking the cuticle can be explained by the presence of pectin projections (hydrophilic pathways) from the cell wall across the cuticular layer, as observed in our study and also reported for nectaries [37,43]. In the case of heterogeneous secretion, the lipophilic portion appears to help in the permeability through the cuticle proper, as inferred by our results in *Allamanda* and *Blepharodon*. Despite these observations, secretion release associated to rupture of the cuticle has been reported in previous studies of some Apocynaceae [12,13,15].

After the secretion reaches the surface, it involves the entire shoot apex and has the function of protecting the meristems [1]. This protection may serve to protect against desiccation due to the hygroscopic character of the mucilage [8,10,44] and against small phytophagous insects, immobilizing them [10]. In addition, lipids produced by colleters are responsible for inhibiting the proliferation of fungi in the meristematic regions [10]. These antifungal lipids play an important role in controlling the growth of fungi since the mucilage present in the secretion of colleters stimulates hyphal growth, as observed in *Peplonia* [10].

## 4. Materials and Methods

Three individuals of each species were collected in São Paulo/SP and in the Parque Estadual da Serra do Mar in Ubatuba/SP. The species chosen for the present study were selected based on the type of secretion produced, according to a previous histochemical study [10] and personal observation, namely *Allamanda schottii* Pohl (Rauvolfioideae) and *Blepharodon bicuspidatum* E.Fourn. (Asclepiadoideae) which produce a heterogeneous secretion, and *Mandevilla splendens* (Hook.f.) Woodson (Apocynoideae) and *Peplonia axillaris* (Vell.) Fontella & E.A.Schwarz. (Asclepiadoideae) which produce a mucilaginous secretion. The vouchers are deposited in the herbaria of the Universidade de São Paulo (SPF: N.V. Capelli 1; J.C. Ribeiro 1) and Universidade Estadual de Campinas (UEC: D. Demarco 7, 35).

### 4.1. Scanning Electron Microscopy

For the micromorphological study, shoot apices were fixed in FAA (formalin, glacial acetic acid, and 50% ethyl alcohol) for 24 h [45], isolated, dehydrated in ethanol series, dried by the critical point method, mounted on aluminum stub, and covered with gold, with subsequent observation in a Jeol JSM 5800 LV scanning electron microscope (Jeol, Tokyo, Japan).

### 4.2. Light Microscopy

For the anatomical analysis, shoot apices and the subsequent nodes were isolated, fixed in BNF (buffered neutral formalin) in 0.1 M sodium phosphate buffer, pH 7.0 [46] for 48 h, dehydrated in a butyl series (tertiary butyl alcohol) [45], embedded in Paraplast (Leica Microsystems Inc., Heidelberg, Germany), and sectioned transversely and longitudinally in a Leica RM2145 rotary microtome. The thickness of the sections ranged from 10 to 12 µm. The sections were stained with astra blue and safranin [47] and the slides mounted in Permount resin (Thermo Fisher Scientific, Waltham, MA, USA). The observations and the photographic records were performed under a Leica DMLB light microscope (Leica Microsystems Inc., Heidelberg, Germany).

### 4.3. Transmission Electron Microscopy

For the ultrastructural study, shoot apices containing colleters at distinct moments of the secretory phase were isolated and fixed in 2.5% glutaraldehyde in 0.1 M sodium phosphate buffer, pH 7.2, postfixed in 1% osmium tetroxide, dehydrated in a graded ketone series, and included in Spurr resin. The sectioning was performed in a Leica Ultracut UCT (Leica Microsystems Inc., Heidelberg, Germany). The ultrathin sections were collected on copper grids (200 mesh) and stained with uranyl acetate [48] and lead citrate [49] with subsequent observation in a Zeiss EM900 transmission electron microscope (Carl Zeiss, Oberkochen, Germany).

For the analysis of the secretory activity, cytochemical tests were performed using ultrathin sections collected on gold grids (200 mesh), facilitating the observation of the interaction between the organelles of the endomembrane system. Ruthenium red was applied for detection of acidic carbohydrates [50], imidazole-buffered osmium tetroxide for lipids [51], and PATAg (periodic acid, thiosemicarbazide and silver proteinate) [52] for polysaccharides. PATAg control test was performed without periodic acid to confirm the presence of polysaccharides.

## 5. Conclusions

Our study found two patterns of secretion in colleters of Apocynaceae, which are related to the composition of secretion: mucilage or a mixture of mucilage and lipophilic compounds. We highlight, as the main novelties, the active role of the vacuole as an organelle of the secretory apparatus, the several intermediate extraprotoplastic secretory storage spaces which arise along the secretion pathway toward the colleter surface, and the cuticular structure which permeability seems to be associated to the proportion of cuticular layer to cuticle proper. The processes described here unveil a diversity of secretion mechanisms in a family with apparently consistent colleter morphology and anatomy, raising new questions for future studies, mainly in relation to the structure of the outer periclinal cell wall and why the passage of secretion through the wall and cuticle eventually forms intramural or subcuticular spaces.
